# SIX3, a tumor suppressor, inhibits astrocytoma tumorigenesis by transcriptional repression of AURKA/B

**DOI:** 10.1186/s13045-017-0483-2

**Published:** 2017-06-08

**Authors:** Zhibin Yu, Yingnan Sun, Xiaoling She, Zeyou Wang, Shuai Chen, Zhiyong Deng, Yan Zhang, Qiang Liu, Qing Liu, Chunhua Zhao, Peiyao Li, Changhong Liu, Jianbo Feng, Haijuan Fu, Guiyuan Li, Minghua Wu

**Affiliations:** 1 Hunan Provincial Tumor Hospital and the Affiliated Tumor Hospital of Xiangya Medical School, Central South University, Changsha, 410013 Hunan China; 2 The Key Laboratory of Carcinogenesis of the Chinese Ministry of Health, The Key Laboratory of Carcinogenesis and Cancer Invasion of the Chinese Ministry of Education, Cancer Research Institute, Central South University, Changsha, Hunan 410008 China; 3 The Second Xiangya Hospital, Central South University, Changsha, Hunan 410011 China; 4 The Third Xiangya Hospital, Central South University, Changsha, Hunan 410011 China; 5 The Xiangya Hospital, Central South University, Changsha, Hunan 410008 China

**Keywords:** SIX3, AURKA, AURKB, Transcriptional repressor

## Abstract

**Background:**

SIX homeobox 3 (SIX3) is a member of the sine oculis homeobox transcription factor family. It plays a vital role in the nervous system development. Our previous study showed that the SIX3 gene is hypermethylated, and its expression is decreased in astrocytoma, but the role of SIX3 remains unknown.

**Methods:**

Chromatin-immunoprecipitation (ChIP) and luciferase reporter assay were used to confirm the binding of SIX3 to the promoter regions of aurora kinase A (AURKA) and aurora kinase B (AURKB). Confocal imaging and co-immunoprecipitation (Co-IP) were used to detect the interaction between AURKA and AURKB. Flow cytometry was performed to assess the effect of SIX3 on cell cycle distribution. Colony formation, EdU incorporation, transwell, and intracranial xenograft assays were performed to demonstrate the effect of SIX3 on the malignant phenotype of astrocytoma cells.

**Results:**

SIX3 is identified as a novel negative transcriptional regulator of AURKA and AURKB, and it decreases the expression of AURKA and AURKB in a dose-dependent manner in astrocytoma cells. Importantly, interactions between AURKA and AURKB stabilize and protect AURKA/B from degradation, and overexpression of SIX3 does not affect these interactions; SIX3 also acts as a tumor suppressor, and it increases p53 activity and expression at the post-translational level by the negative regulation of AURKA or AURKB, reduces the events of numerical centrosomal aberrations and misaligned chromosomes, and significantly inhibits the proliferation, invasion, and tumorigenesis of astrocytoma in vitro and in vivo. Moreover, experiments using primary cultured astrocytoma cells indicate that astrocytoma patients with a low expression of SIX3 and mutant p53 are more sensitive to treatment with aurora kinase inhibitors.

**Conclusion:**

SIX3 is a novel negative transcriptional regulator and acts as a tumor suppressor that directly represses the transcription of AURKA and AURKB in astrocytoma. For the first time, the functional interaction of AURKA and AURKB has been found, which aids in the protection of their stability, and partially explains their constant high expression and activity in cancers. SIX3 is a potential biomarker that could be used to predict the response of astrocytoma patients to aurora kinase inhibitors.

**Electronic supplementary material:**

The online version of this article (doi:10.1186/s13045-017-0483-2) contains supplementary material, which is available to authorized users.

## Background

Malignant astrocytomas are the most common and aggressive malignancy of the central nervous system, especially glioblastoma (GBM). Individuals with GBM have a median survival of 15 months and only a 27% 2-year patient survival rate due to the lack of effective therapeutic options [1]. Chromosomal instability (CIN) is one of the hallmarks of the cancer, and its causative role in tumorigenesis has been recognized for over a century [2]. Complex mechanisms of CIN have been explored and could provide attractive therapeutic targets in cancer [3]. Dozens of genes are involved in ensuring chromosomal stability. Mutation and misexpression of these genes are often an early event in tumor initiation, resulting in aneuploidy and heterogeneity of cancer cells [4].

The aurora kinase family is a collection of highly related serine/threonine kinases that are key regulators of mitosis. Defects in these kinases lead to severe mitotic abnormality that induces CIN [5]. Three members of the aurora kinase family, namely, aurora kinase A (AURKA), aurora kinase B (AURKB), and aurora kinase C (AURKC), have been found in mammalian cells [5, 6]. AURKA and AURKB are expressed in somatic cells, and AURKC is limited to germ cells that undergo meiosis, though its function overlaps with that of AURKB [7]. Aurora kinases possess a conserved catalytic domain and divergent N-termini that determine the different sub-cellular localization of each aurora kinase [6, 7]. AURKA is located in centrosomes, spindle microtubules, and the central spindle and is required for centrosome maturation, mitotic entry, and centrosome separation [6, 8, 9]. AURKB is located within centrosomes where it constitutes the chromosome passenger complex (CPC), and in the midzone/midbody, regulating spindle assembly checkpoint and the kinetochore [6, 10].

The overexpression of aurora kinases is found in a large number of human cancers [5, 11]. The ectopic expression of AURKA and AURKB is closely correlated with carcinogenesis [12], the epithelial-to-mesenchymal transition [13], cancer cell stemness [14], and drug resistance [15]. Targeting aurora kinases is being investigated as a potential therapy, and several pan-aurora kinase inhibitors or selective aurora kinase inhibitors have been introduced in clinical trials of human cancers [16, 17]. The transcription of AURKA and AURKB is regulated in a cell cycle-dependent manner. The promoter regions of these genes contain a cell cycle-dependent element (CDE) and a cell cycle gene homology region (CHR), where transcription factors E2F1, E2F4, and DP2 bind [18, 19]. The oncoprotein c-Myc has been found to directly activate the transcription of AURKA via E-boxes and to indirectly activate the transcription of AURKB in Myc-driven B cell lymphoma [20]. However, the suppressive mediator of aurora kinase has not been well studied.

The RDGN (retinal determination gene network) is a well-conserved regulatory network, which consists of a series of transcription factors or cofactors that are involved in the formation of multiple organs including the eye, brain, kidney, and muscle [21]. The Six family, as well as Pax, Eya, and Dach, are mainly members of RDGN, and they function independently or cooperatively with cofactors and transcriptionally regulate downstream signaling of cell differentiation and proliferation. The misexpression or mutation of these genes is found to be associated with numerous human diseases, including human cancers [21–23]. The RDGN genes have been found in numerous essential pathways that are involved in tumor initiation and progression. In breast cancer, SIX1 activates extracellular signal-regulated kinase and transforming growth factor-beta signaling pathways, which result in the transformation of mammary epithelial cells and increase the proliferation of cancer stem cells (CSCs) [24, 25]. Another member of the RDGN, Dach1, has been found to exert tumor-suppressive role through cell cycle arrest by interacting with p53 and to inhibit CSCs and TGF-β/SMAD-induced epithelial-mesenchymal transition [26, 27]. SIX homeobox 3 (SIX3) is a member of the Six transcription factor family and contains a homeobox domain for DNA binding to DNA and a six domain [23]. SIX3 is predominantly expressed in the CNS (central nervous system) and is critical for CNS development [28–31]. Mutations of SIX3 have been found to correlate with multiple CNS developmental disorders, such as holoprosencephaly, aprosencephaly, and atelencephaly in humans [32–34]. The ectopic expression of SIX3 has also been observed in several human cancers, although it may have a dual effect on cancers [35–37]. SIX3 has been found to be upregulated in non-small cell lung cancer and correlated with higher TNM stages [36]. While in lung adenocarcinoma, SIX3 is downregulated and indicates better prognosis [35]. Restoration of SIX3 suppresses cell proliferation and migration of lung adenocarcinoma [35]. We have previously found SIX3 to be hypermethylated in human astrocytoma [38], but the exact role of SIX3 in astrocytoma tumorigenesis remains unknown.

## Results

### SIX3 is a transcriptional repressor of AURKA and AURKB

**Fig. 1:**
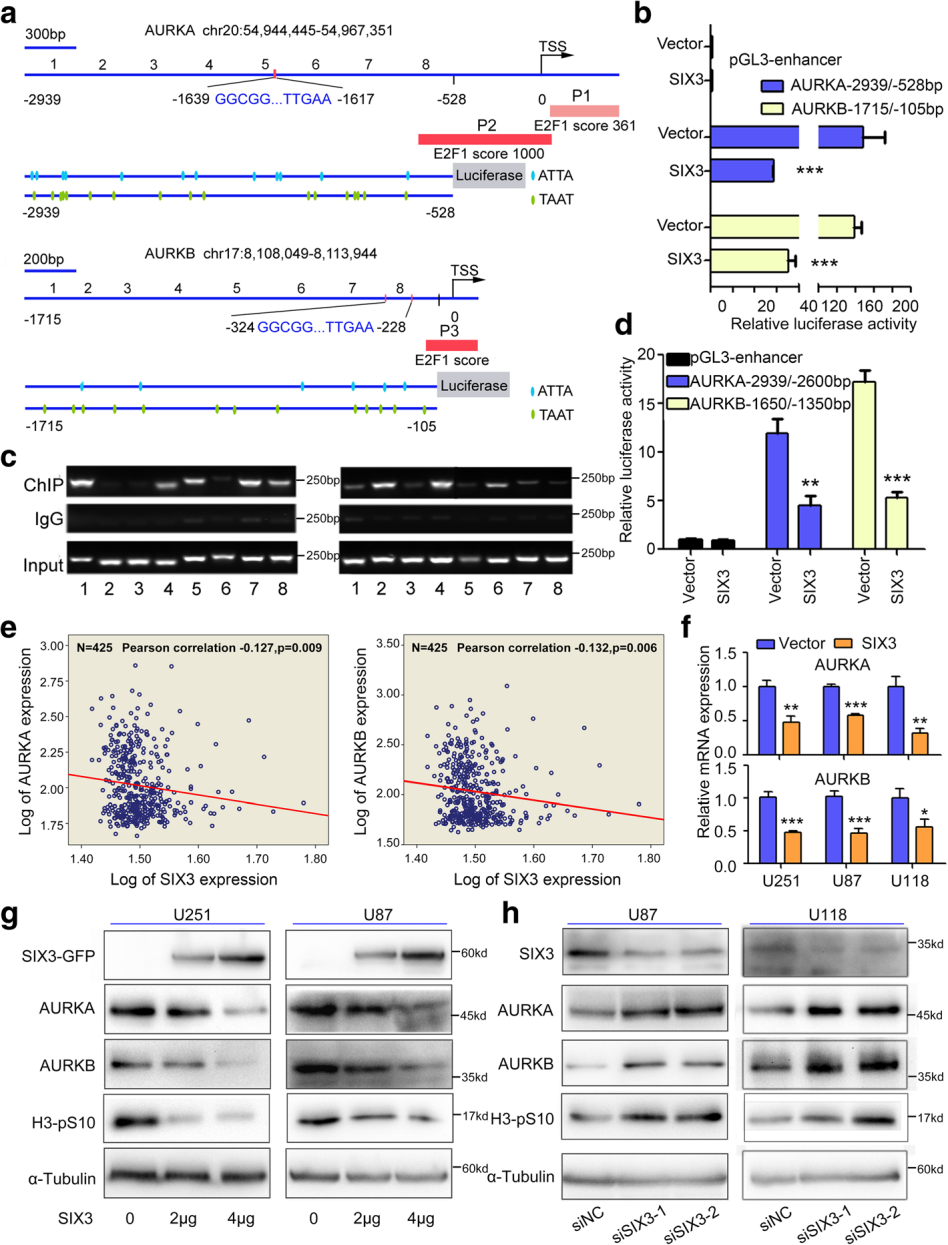
SIX3 directly suppresses the transcription activity of AURKA and AURKB. **a** Schematic diagrams of promoters of AURKA and AURKB. TSS = 0; the numbers 1–8 refer to regions for ChIP assay; P1, P2, and P3 represent E2F1-binding regions, and the scores are the ChIP-seq data from the ENCODE project. **b** Promoter regions of AURKA (−2939 to −528 bp) and AURKB (−1715 to −105 bp) were inserted into pGL3-enhancer vector and co-transfected with pEGFP-C1-SIX3 into U251 cells. Luciferase reporter assay indicated that SIX3 suppressed the transcriptional activity of AURKA and AURKB promoters. **c** U251 cells were transfected with p3xFLAG-CMV-10-SIX3 plasmid. Anti-Flag antibody was used for immunoprecipitation. ChIP assay showed differential recruitment of SIX3 to the promoter regions of AURKA and AURKB. Immunoprecipitated chromatin was subjected to PCR and amplified for 29 cycles according to the data from RT-qPCR and then analyzed by agarose gel electrophoresis. The numbers 1–8 correspond to the numbers in the schematic diagram in **a. d** Promoter regions of AURKA (−2939 to −2600 bp) and AURKB (−1650 to −1350 bp) were inserted into pGL3-enhancer vector and co-transfected with pEGFP-C1-SIX3 into U251 cells. Luciferase reporter assays indicated that SIX3 suppressed the transcriptional activity of AURKA and AURKB promoters. **e** Analysis of REMBRANDT showed inverse correlation between SIX3 and AURKA and SIX3 and AURKB in glioma. **f** U251, U87, and U118 cells were transfected with pEGFP-C1-SIX3. RT-qPCR showed that SIX3 reduced the mRNA level of AURKA and AURKB (normalized with GAPDH). **g** U251 and U87 cells were transfected with different doses of pEGFP-C1-SIX3. Western blotting analysis showed that SIX3 decreased the expression of AURKA, AURKB, and phospho-histone 3 (H3-pS10) in a dose-dependent manner. **h** U87 and U118 cells were transfected with two different siRNAs targeting SIX3. Western blotting analysis showed that the knockdown of SIX3 increased the expression of AURKA, AURKB, and phospho-histone 3 (H3-pS10) (*P < 0.05; **P < 0.01; ***P < 0.001)

In addition to the positive regulatory regions identified by Tanaka [19] (AURKA −124 to −75 bp, TSS = 0) and Kimura [18] (AURKB −104 to −74 bp), we analyzed the promoter regions of AURKA from −2939 to −528 bp and AURKB from −1715 to −105 bp (Fig. 1a), which cover the regulatory regions of the respective genes according to the data from the Encyclopedia of DNA Elements (ENCODE) project [39]. Known CDE-CHR regions were found in both AURKA (−1617 to −1636 bp, Fig. 1a) and AURKB (−228 to −324 bp, Fig. 1a). Additionally, there were two AT-rich regions thought to be associated with regulatory elements [40]. Abundant ATTA/TAAT repeating sequences were found among these regions (Fig. 1a). Intriguingly, the ATTA/TAAT sequence coincides with the core motif recognized by SIX3 [30, 41]. To confirm whether SIX3 regulates the transcriptional activity of the promoter regions of AURKA and AURKB, SIX3-expressing plasmid (Additional file 1: Figure S1) and AURKA/AURKB luciferase reporters were co-transfected into U251 cells. SIX3 expression significantly decreased the promoter activity of AURKA and AURKB (Fig. 1b). To examine the physical binding of SIX3 with the promoters of AURKA and AURKB, a chromatin-immunoprecipitation (ChIP) assay was carried out with primers specifically designed against every 300 bp sequence of the AURKA promoter (−2939 to −528 bp) and every 200 bp sequence of AURKB promoter (−1715 to −105 bp) (Fig. 1a). The results of this experiment demonstrated that SIX3 bound to regions 1, 5, and 7 of the AURKA promoter and to regions 2 and 4 of the AURKB promoter (Fig. 1c). These regions contain higher densities of ATTA/TAAT repeating sequences except in region 4 of the AURKB promoter. Since region 1 of the AURKA promoter and region 2 of the AURKB promoter had the highest level of SIX3 binding as well as density of ATTA/TAAT repeating sequence, we cloned these two regions to assess the AURKA/AURKB promoter activity. The overexpression of SIX3 significantly suppressed the activity of these regions of the AURKA and AURKB promoters (Fig. 1d).

The analysis of Repository for Molecular Brain Neoplasia Data (REMBRANT) revealed strong negative correlation between SIX3 and AURKA and between SIX3 and AURKB in glioma (Fig. 1e). The expression of SIX3, AURKA, and AURKB were further studied in 18 human astrocytoma samples, and both AURKA and AURKB expression showed an inverse relationship with the expression of SIX3 (Additional file 1: Figure S2). A strong inverse correlation between SIX3 and AURKA/AURKB were obtained with normal astrocyte cell line HEB and three astrocytoma cell lines by Western blotting (Additional file 1: Figure S3). Overexpression of SIX3 decreased the expression of AURKA and AURKB in a dose-dependent manner (Fig. 1f, g). Knockdown of SIX3 with two independent small interference RNAs (siRNAs) increased the expression of AURKA and AURKB (Fig. 1h and Additional file 1: Figure S4). A common downstream effect of AURKA and AURKB activity, histone H3 phosphorylation on Ser10, was detected. Our results showed that the overexpression of SIX3 decreased H3 phosphorylation, while knockdown of SIX3 increased H3 phosphorylation (Fig. 1g, h).

The above data indicate that SIX3 acts as a transcriptional repressor of AURKA and AURKB, suppressing the transcription of AURKA and AURKB by directly binding with their promoters in astrocytoma.

### SIX3 reactivates the p53 pathway mainly through AURKA and AURKB

**Fig. 2:**
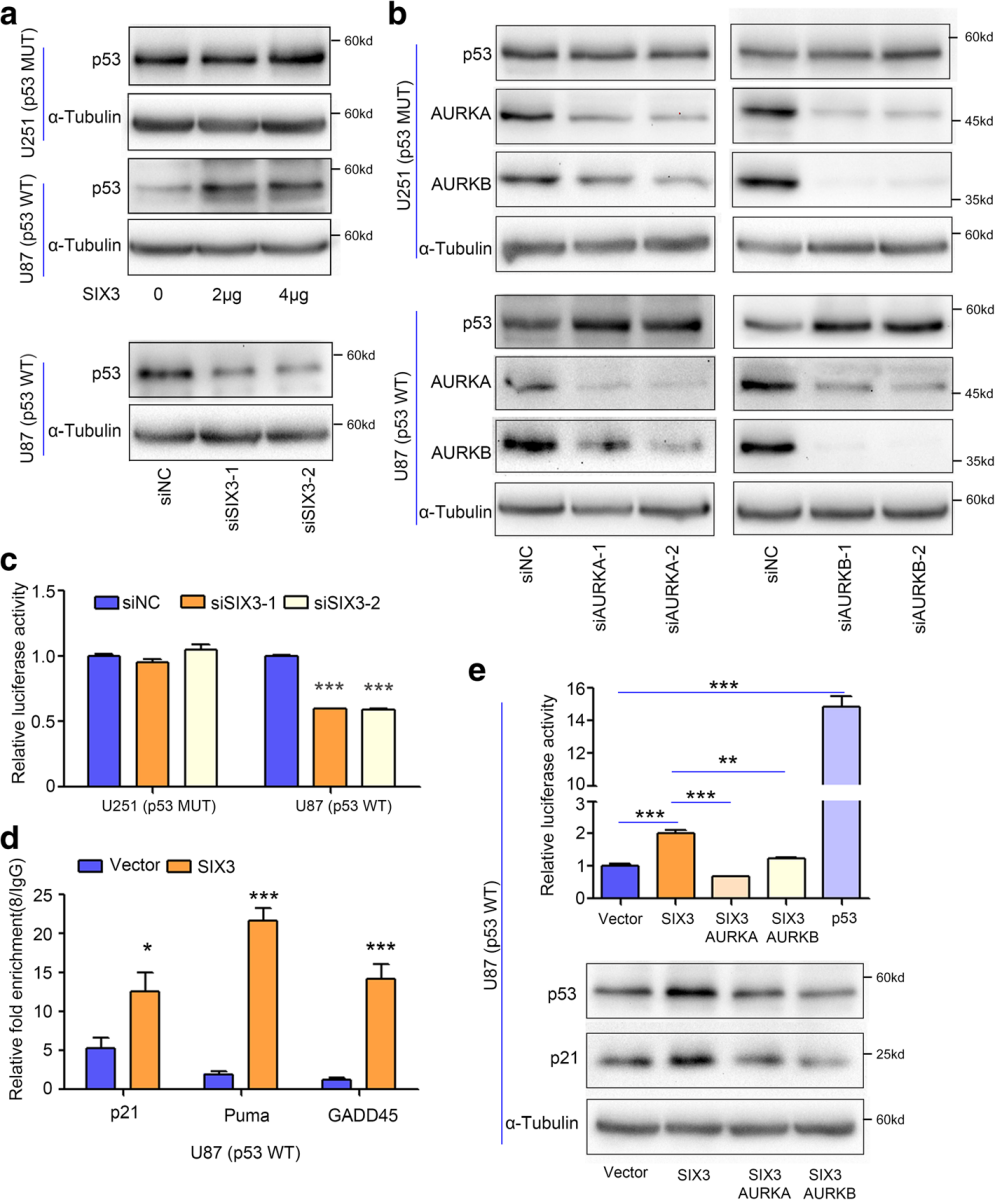
SIX3 reactivates the p53 pathway dependent on suppressing of AURKA and AURKB. **a** U251 and U87 cells were transfected with pEGFP-C1-SIX3 and siRNAs targeting SIX3. Western blotting analysis showed that SIX3 did not affect p53 expression in p53 mutant U251 cells. Overexpression of SIX3 increased p53 expression, and knockdown of SIX3 reduced p53 expression in p53 wild-type U87 cells. **b** U251 and U87 cells were transfected with two different siRNAs targeting AURKA and AURKB. Western blotting analysis showed that knockdown of AURKA or AURKB did not affect p53 expression in p53 mutant U251 cells but increased p53 expression in p53 wild-type U87 cells. **c** p53 activity reporter and siRNAs targeting SIX3 were co-transfected into p53 wild-type U87 cells. Luciferase activity analysis showed that knockdown of SIX3 expression decreased the activity of p53 in p53 wild-type U87 cells. **d** U87 cells were transfected with pEGFP-C1-SIX3. ChIP assay indicated overexpression of SIX3 enhanced the recruitment of p53 protein to the promoter regions of well-known p53 targets, including p21, PUMA, and GADD45. Chromatin samples were subjected to RT-qPCR. **e** U87 cells were transfected with pEGFP-C1-SIX3, pEGFP-C1-SIX3 and p3xFlag-CMV-10-AURKA, and pEGFP-C1-SIX3 and p3xFlag-CMV-10-AURKB, respectively, and pEGFP-C1-p53 was the positive control. Luciferase activity analysis and Western blot showed that co-transfection of AURKA or AURKB with SIX3 counteracted the effect of SIX3 on increasing the expression and activity of p53 in U87 cells (*P < 0.05; **P < 0.01; ***P < 0.001)

The tumor suppressor gene p53 acts as a guardian of the genome and plays a vital role in multiple cellular processes [42]. AURKA and AURKB have been found to directly phosphorylate p53 and accelerate the degradation of p53 through Mdm2-mediated ubiquitination, promoting carcinogenesis and cancer progression [43, 44]. We found that SIX3 overexpression increased p53 expression in p53 wild-type U87 cells and did not affect p53 expression in p53 mutant U251 cells (Fig. 2a). Moreover, knockdown of SIX3 suppressed p53 expression in p53 wild-type U87 (Fig. 2a). We further knocked down the expression of AURKA or AURKB and found that the knockdown of AURKA or AURKB led to an upregulation of p53 in p53 wild-type U87 but did not affect the expression of p53 in p53 mutant U251 cells (Fig. 2b). We also found no effect of SIX3 on the messenger RNA (mRNA) expression of p53 (Additional file 1: Figure S5).

Subsequently, we detected the effect of SIX3 on p53 activity. Knockdown of SIX3 dramatically decreased p53 activity in p53 wild-type U87 (Fig. 2c). A ChIP assay confirmed the enhanced enrichment of p53 on the promoters of well-known p53 target genes p21, Puma, and GADD45 when SIX3 was overexpressed in p53 wild-type U87 cells (Fig. 2d). The rescue of AURKA or AURKB expression reversed the effect of SIX3 on the expression and activity of p53 in p53 wild-type U87 cells (Fig. 2e).

Altogether, these data indicate that SIX3 increases p53 activity and expression at the post-translational level by negative regulation AURKA or AURKB.

### Interaction between AURKA and AURKB which protects them from degradation is not affected by SIX3

**Fig. 3:**
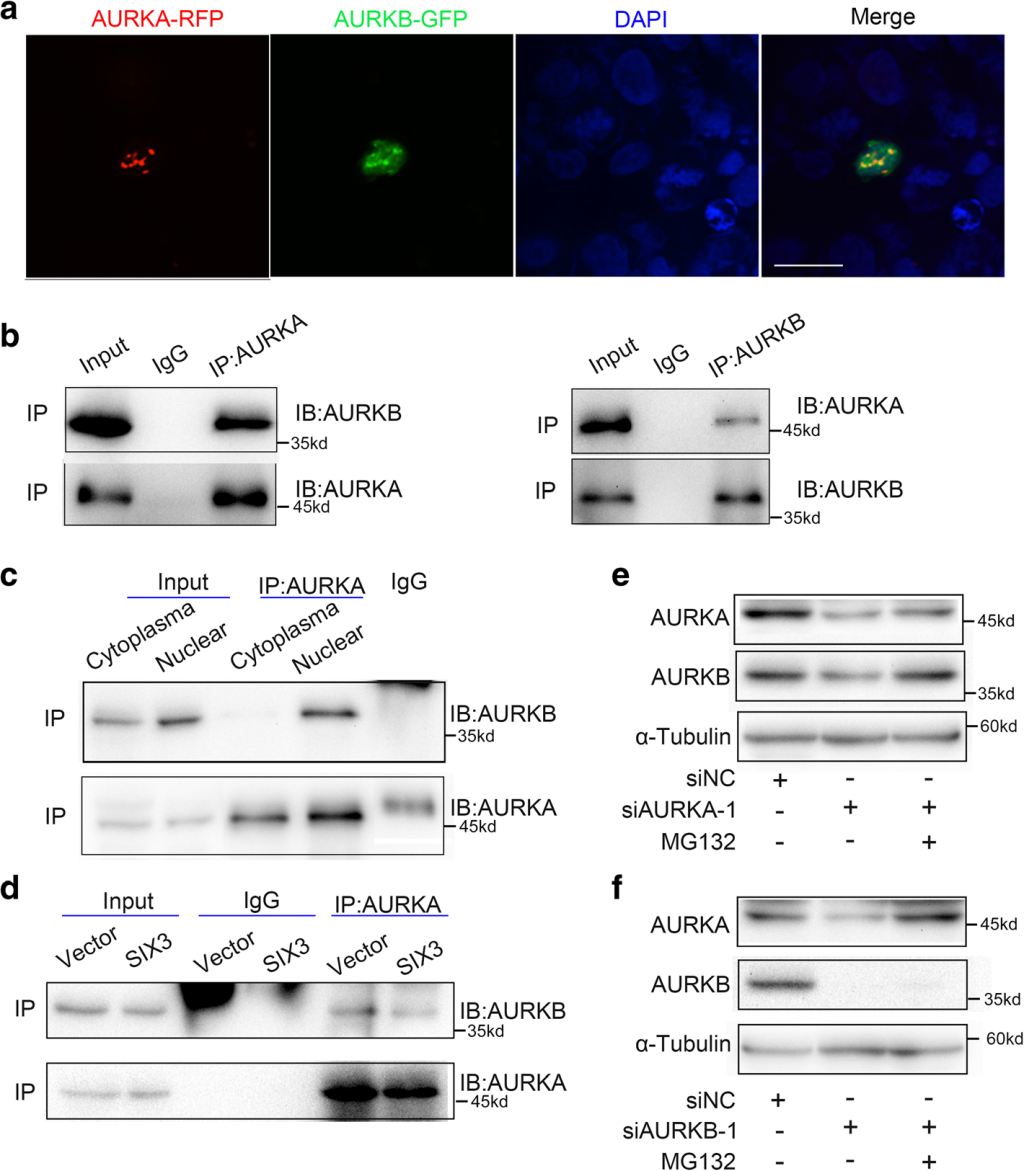
Interactions between AURKA and AURKB protects them from degradation. **a** Representative confocal images of HEK293 cells co-transfected with AURKA-GFP (*green*) and AURKB-RFP (*red*). The merged images showed co-localization of AURKA and AURKB in the nuclei. (*scale bars*, 29 μm). **b** Co-IP and Western blotting analysis showed the interaction between endogenous AURKA and AURKB in U251 cells. **c** Nuclear and cytoplasmic proteins were extracted from U251 cells; IP and Western blotting analysis demonstrated interaction between endogenous AURKA and AURKB mainly in the nuclear fractions. **d** U251 cells were transfected with pEGFP-C1-SIX3. IP and Western blotting analysis showed that SIX3 did not affect the interaction between AURKA and AURKB but decreased the amount of interacting AURKA and AURKB. **e**, **f** U251 cells was transfected with siRNAs targeting AURKA. Twenty-four hours later, the U251 cells transfected with siRNAs were treated with MG132 (20 μM) for another 2 h. Western blotting analysis showed that MG132 treatment recovered AURKB protein level upon AURKA knockdown (**e**) and recovered AURKA protein level upon AURKB knockdown (**f**)

As shown in Fig 2b, an unexpected reduction in AURKB expression was found upon knockdown of AURKA, and reduction in AURKA expression was also found upon knockdown of AURKB (Fig. 2b). After ruling out off-target effects of siRNAs by detecting the mRNA levels of AURKA and AURKB after transfection with independent siRNAs (Additional file 1: Figure S6), we further showed the nuclear co-localization of green fluorescent protein (GFP)-tagged AURKA and red fluorescent protein (RFP)-tagged AURKB in HEK293 cells (Fig. 3a). Co-immunoprecipitation (co-IP) analysis further validated the interaction between endogenous AURKA and AURKB (Fig. 3b). Moreover, consistent with the observed co-localization by confocal microscopy, co-IP showed the interaction of AURKA and AURKB mainly in the nuclear fraction of U251 cells (Fig. 3c). We used co-IP to further authenticate the interaction between AURKA and AURKB when AURKA-Flag and AURKB-GFP were co-transfected in U251 cells (Additional file 1: Figure S7). Our results indicated that SIX3 had no influence on the interaction between AURKA and AURKB, but the overexpression of SIX3 decreased the amount of AURKB immunoprecipitated by the AURKA antibody, which is likely due to the negative transcriptional regulation of SIX3 on the promoter of AURKA and AURKB (Fig. 3d).

AURKA and AURKB are primarily degraded through the anaphase-promoting complex ubiquitin-proteasome pathway [45]. We wanted to know whether the cross talk between AURKA and AURKB depends on proteasome activity. U251 cells were treated with MG132 to block ubiquitin-proteasome-mediated protein degradation after transfection with siRNAs targeting AURKA or AURKB. When we knocked down AURKA, MG132 resulted in increased AURKB expression and had less effect on AURKA (Fig. 3e). The inverse phenomenon occurred when we knocked down AURKB expression (Fig. 3f). Thus, the interaction between AURKA and AURKB helps to protect both proteins from degradation, which partially explains the tight correlation between the expression of these two aurora kinases in tumors.

### SIX3 induces S phase arrest independent of AURKA and AURKB

**Fig. 4:**
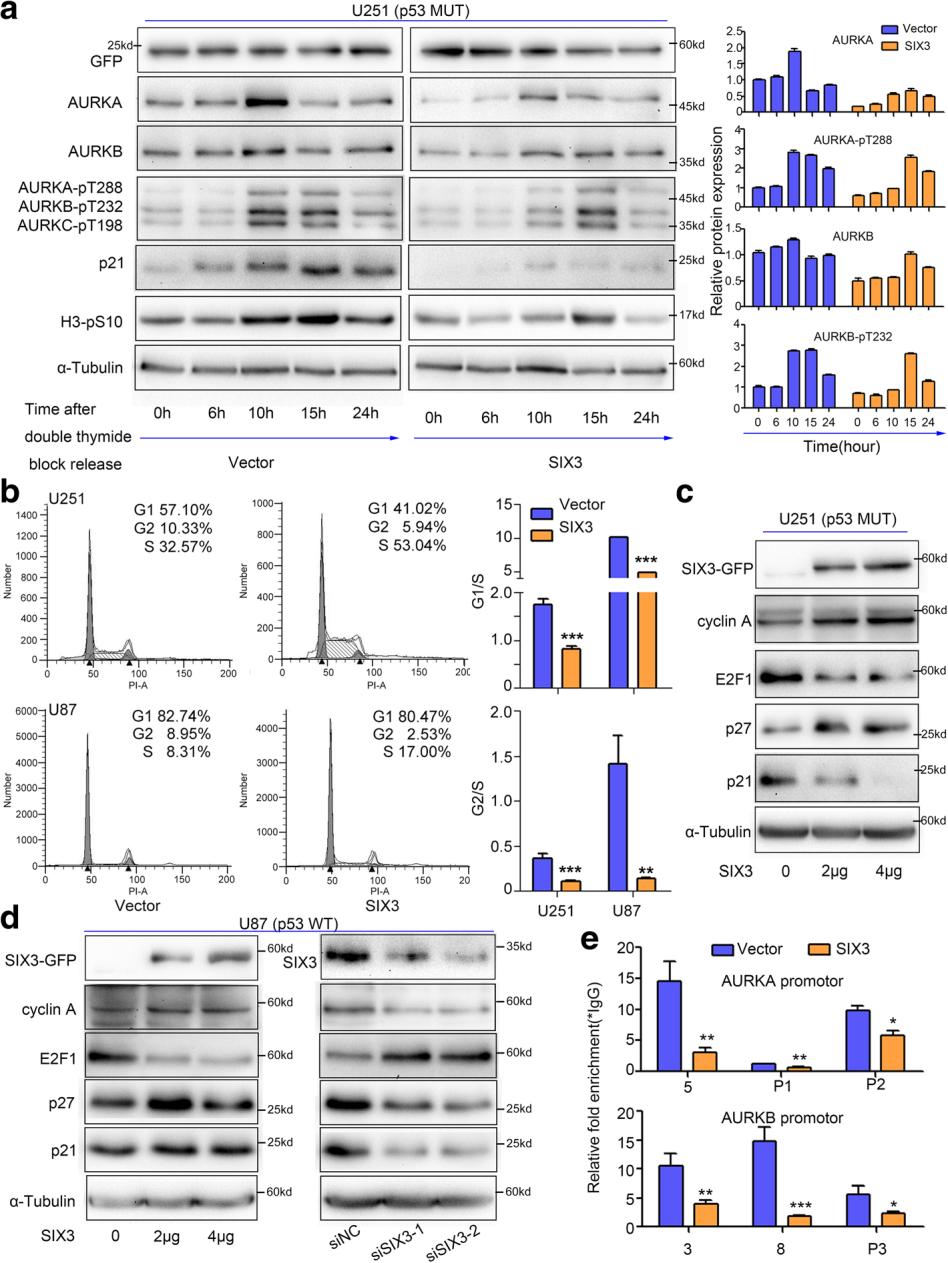
SIX3 induces S phase arrest and reduces the recruitment of E2F1 on the promoters of AURKA and AURKB. **a** U251 cells were synchronized by double thymidine block and transfected with pEGFP-C1-SIX3 24 h before release. Western blotting analysis showed that SIX3 decreased the expression of AURKA, AURKB, phospho-AURKA (Thr288)/AURKB (Thr232)/AURKC (Thr198), phospho-histone 3 (S10), and p21 and delayed the aurora kinase peak approximately 5 h. **b** Flow cytometry showed that SIX3 induced S phase arrest in both U251 and U87 cells. Quantification of G1/S and G2/S ratios are shown on the *right*. **c** Western blotting analysis showed that SIX3 increased the expression of cyclin A and p27 and decreased the expression of E2F1 and p21 in U251 cells. **d** U87 cells were transfected with pEGFP-C1-SIX3 and siRNAs targeting SIX3. Western blotting analysis showed that SIX3 increased the expression of cyclin A, p27, and p21 and decreased expression of E2F1 (*left*). Knockdown of SIX3 increased the expression of E2F1 and decreased expression of cyclin A, p27, and p21. **e** ChIP assay indicated that SIX3 decreased the enrichment of E2F1 on the promoter regions of AURKA and AURKB in U251 cells. Chromatin samples were subjected to RT-qPCR analysis (*P < 0.05; **P < 0.01; ***P < 0.001)

AURKA and AURKB are cell cycle-regulated genes and have similar expression profiles as they share the same transcriptional regulators [18, 19]. To investigate the suppressive effect of SIX3 on the expression of AURKA and AURKB throughout the cell cycle, we synchronized U251 cells using double thymidine block to block cells at G1/S phase (Additional file 1: Figure S10). Then, we overexpressed SIX3 in the U251 cells before releasing them and observed the dramatically reduced expression of AURKA and AURKB (Fig. 4a). A previous study showed that the expression and activation of AURKA and AURKB begin in S phase and peak in the G2/M phase [11]. Surprisingly, we found that in the SIX3-overexpressing cells, aurora kinase activation peaked 5 h later than in the control cells, suggesting that SIX3 prolongs the G1/S phase (Fig. 4a). Flow cytometric analysis showed that SIX3 induces S phase arrest both in p53 wild-type U87 cells and p53 mutant U251 cells (Fig. 4b), and the restoration of AURKA or AURKB could not reverse the SIX3-induced S phase arrest (Additional file 1: Figure S9, S10). We also found that the knockdown of AURKB blocked the U251 and U87 cells in the G2/M phase (Additional file 1: Figures S10 and S11). However, the knockdown of AURKA arrested p53 mutant U251 cells in G1 phase (Additional file 1: Figure S10), whereas it arrested the p53 wild-type U87 cells in G2/M phase (Additional file 1: Figure S11). The above data suggested that the S phase arrest caused by SIX3 was not correlated with p53, AURKA, or AURKB, although SIX3 increased the activity of p53 via the negative regulation of AURKA or AURKB.

Next, we attempted to elucidate the mechanism of S phase arrest induced by SIX3. Several factors involved in the G1/S phase regulation were analyzed. The overexpression of SIX3 increased the expression of cyclin A and p27 and decreased the expression of E2F1 in both U251 (Fig. 4c) and U87 cells (Fig. 4d). The knockdown of SIX3 significantly upregulated the expression of E2F1 and downregulated the expression of cyclin A and p27 (Fig. 4d). E2F1 is a transcriptional activator promoting the transition from the G1 phase to the S phase [46] and also has been found to be a transcriptional activator of AURKA and AURKB [18, 19]. Our studies confirmed the recruitment of E2F1 to the promoters of AURKA and AURKB. Concordant with the scores provided by ENCODE [39], E2F1 mostly assembled at P2 and P3 rather than at P1 (Figs. 1a and 4e). In addition, regions containing CDE-CHR (region 5 of AURKA and region 8 of AURKB) or E2F1-binding motif GGCGG (region 3 of AURKB) were found to bind with E2F1 as well (Figs. 1a and 4e). The overexpression of SIX3 decreased the expression of E2F1 and reduced the enrichment of E2F1 on the promoters of AURKA or AURKB (Fig. 4e).

The above data indicate that the SIX3-induced S phase arrest is independent of p53, AURKA, and AURKB but is correlated with the regulation of cyclin A, E2F1, and p27.

### SIX3 reduces numerical centrosomal aberrations and inhibits astrocytoma tumorigenesis

**Fig. 5:**
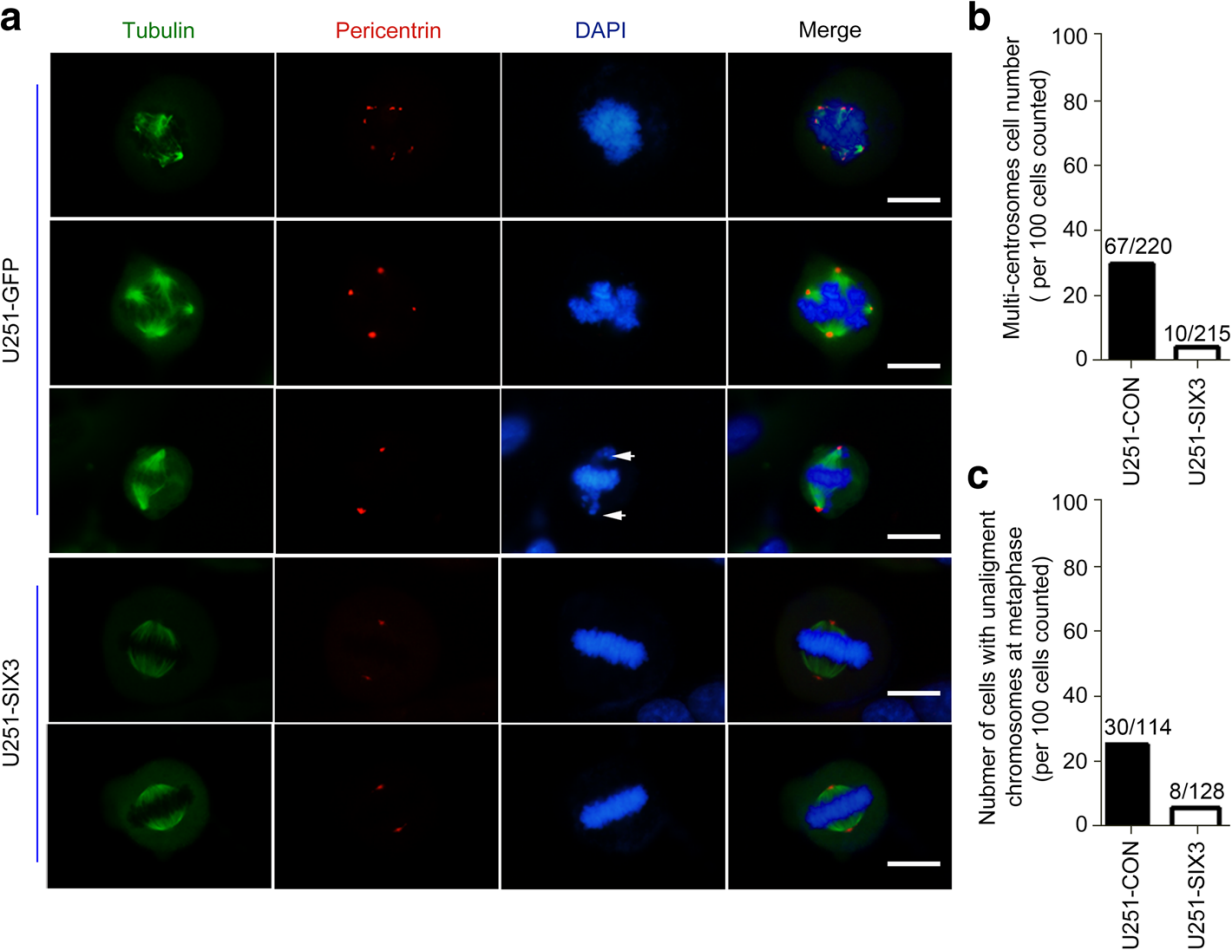
SIX3 reduces numerical centrosomal aberrations. U251 cells stably expressing SIX3 (U251-SIX3) and U251 cells stably expressing GFP (U251-CON) were constructed using lenti-virus. U251-CON and U251-SIX3 cells were synchronized with double thymidine block and blocked at M phase with MG132 (20 μM) for 2 h. **a** Immunofluorescence analysis showed typical multi-centrosomes and chromosome unalignment (*white arrows*) in U251-CON and U251-SIX3 cells (*scale bars*, 20 μm). **b** Centrosomes in more than 200 cells were analyzed. **c** More than 100 cells were observed for unaligned chromosomes

AURKA and AURKB are key regulators of mitosis and are associated with centrosome maturation and separation, regulating chromatin modification and cytokinesis [5]. We thus generated U251 cells stably expressing SIX3 (U251-SIX3) and U251 cells stably expressing GFP (U251-CON) using lenti-virus and sorted them by flow cytometry (Additional file 1: Figure S12). More than 200 U251-SIX3 and U251-CON cells in the M phase were investigated. We found that SIX3 overexpression reduced the percentage of three or more centrosomes (4.65%, 10/215), compared with the control cells (30.45%, 67/220) (Fig. 5a, b), and SIX3 overexpression also decreased the incidence of misaligned chromosomes at metaphase (6.25%, 8/128), compared with control cells (26.32%, 30/114) (Fig. 5a, c).

**Fig. 6:**
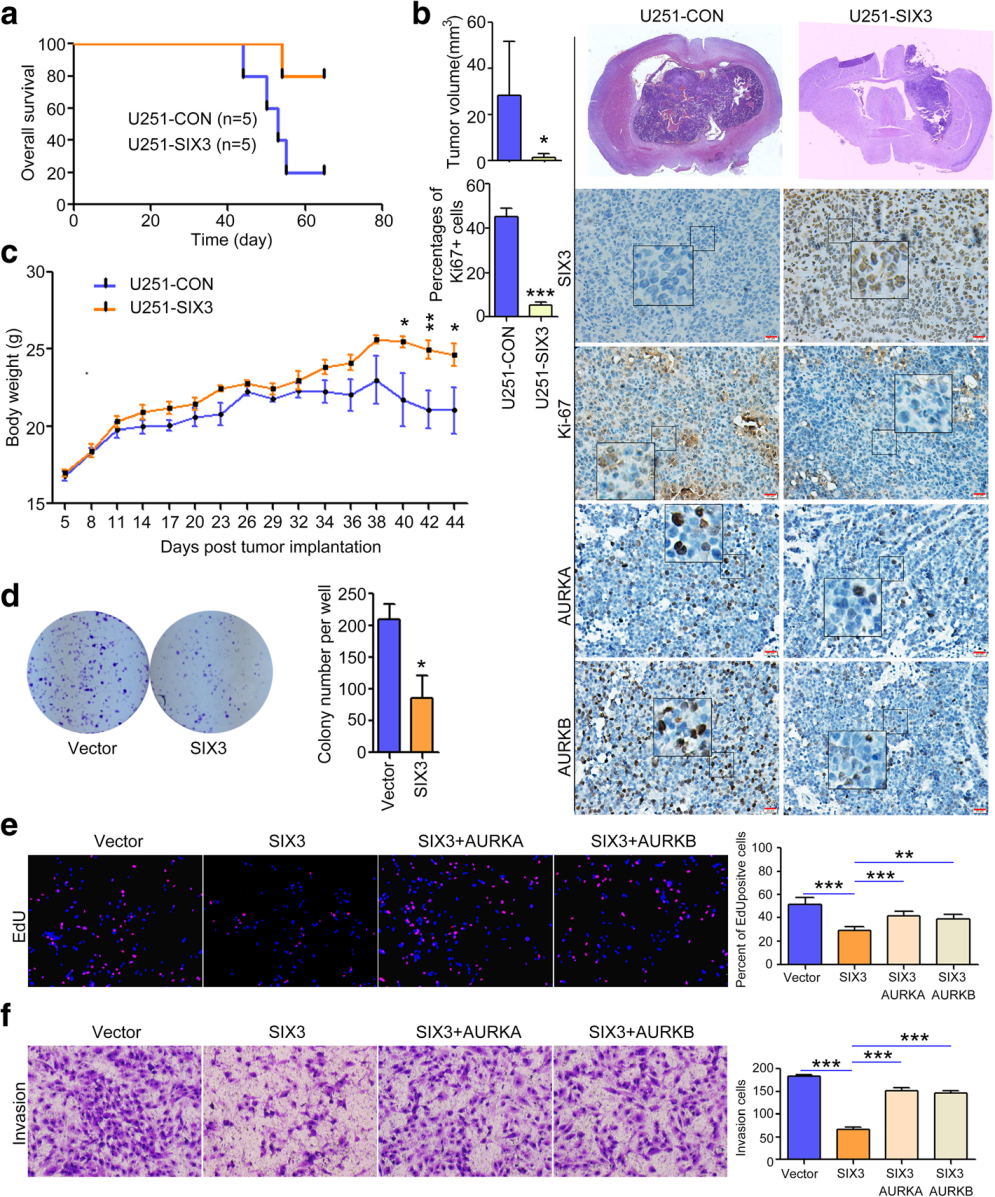
SIX3 inhibits the astrocytoma tumorigenesis in vivo and in vitro. **a** U251-CON and U251-SIX3 cells were intracranially injected into the brains of nude mice. Survival analysis showed that mice transplanted with U251-SIX3 cells exhibited longer survival compared to mice transplanted with U251-CON cells. **b** H&E staining and immunohistochemistry analysis showed that mice transplanted with U251-SIX3 cells exhibited smaller tumors and lower expression of Ki-67, AURKA, and AURKB. **c** The growth curves measuring weight in the two groups of mice. When mice began to die, we stopped recording their weight. **d** U251 cells were transfected with pEGFP-C1-SIX3. Colony formation assay showed that SIX3 reduced the proliferation of U251 cells. **e**, **f** U251 cells were transfected with pEGFP-C1-SIX3, SIX3 and AURKA, and SIX3 and AURKB. EdU incorporation and transwell assay showed that rescue of AURKA and AURKB reversed the suppressive effect of SIX3 on proliferation (**e**) and invasion (**f**) of U251 cells (*P < 0.05; **P < 0.01; ***P < 0.001)

We further identified the role of SIX3 on GBM tumorigenesis. U251-SIX3 and U251-CON cells were intracranially injected into nude mice. Compared to mice transplanted with U251-CON cells, mice transplanted with U251-SIX3 cells exhibited longer survival (Fig. 6a) and smaller tumors (Fig. 6b) and gained more weight by the end of the experiment (Fig. 6c). Overexpression of SIX3 significantly decreased the expression of Ki-67, AURKA, and AURKB (Fig. 6b).

SIX3 also notably suppressed the proliferation (Fig. 6d, e) and invasion of astrocytoma cells (Fig. 6f). On this basis, the overexpression of AURKA or AURKB significantly abrogated the effect of SIX3 overexpressison on suppressing cell proliferation and invasion (Fig. 6e, f). The above data indicate that SIX3 exerts a tumor-suppressive effect, and restoration of SIX3 can reverse the malignant phenotype of human astrocytoma.

### SIX3 expression indicates the sensitivity of astrocytoma cells to aurora kinase inhibitors

**Fig. 7:**
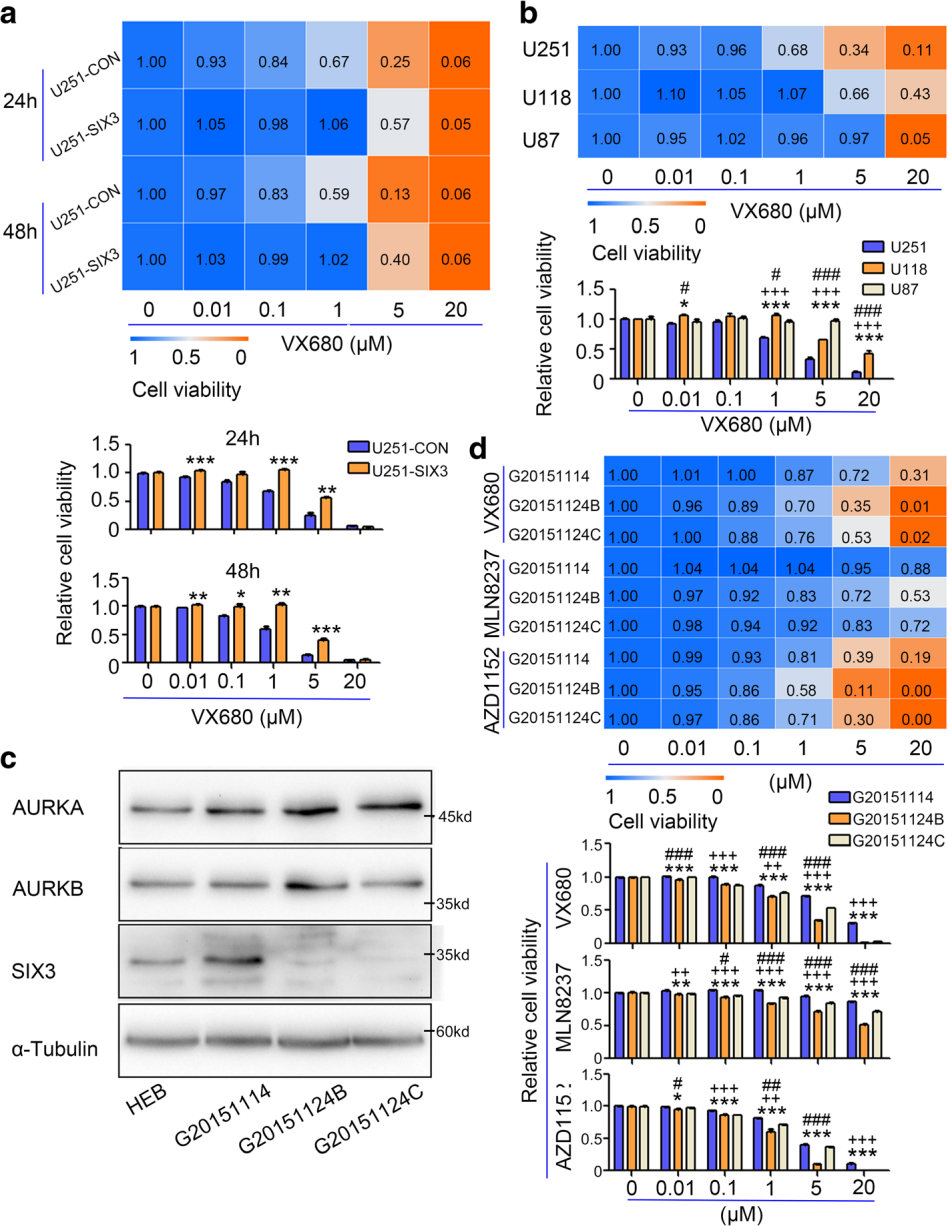
SIX3 expression indicates the sensitivity of astrocytoma cells to aurora kinase inhibitors. **a** U251-CON and U251-SIX3 cells were treated with a concentration gradient of VX680 for 48 and 72 h and subjected to CCK8 assay. The data represent the mean of five replicates. **b** U251 and U87 cells were treated with a concentration gradient of VX680 for 48 h and subjected to CCK8 assay. The data represent the mean of five replicates. * U251 vs U118, + U251 vs U87, # U118 vs U87. **c** Western blotting analysis showed the expression of SIX3, AURKA, and AURKB in normal astrocytes and three primary human patient-derived astrocytomas. **d** The three primary patient-derived astrocytomas were treated with a concentration gradient of VX680, MLN8237, and AZD1152 for 48 h and subjected to CCK8 assays. * G20151104 vs G20151124B, + G20151104 vs G20151124C, # G20151124B vs G20151124C. The data represent the mean of five replicates (* or + or #, *P* < 0.05; ** or ++ or ##, *P* < 0.01; *** or +++ or ###, *P* < 0.001)

Several studies have reported the potential of using aurora kinase inhibitors in the treatment of brain tumors. One such study found that radiotherapy followed by VX680 treatment reduced growth and increased survival of GBM models [47, 48]. Several genetic biomarkers, such as AURKA, p53, and c-Myc, have been found to regulate the response to aurora kinase inhibitors, [20, 49, 50]. Since SIX3 has been confirmed as a negative transcriptional regulator of AURKA and AURKB, we wondered whether SIX3 expression is correlated with the sensitivity of astrocytoma cells to aurora kinase inhibitors. We found that the U251-SIX3 cells showed less sensitivity to VX680 compared with the U251-CON cells (Fig. 7a). With increased expression of AURKA or AURKB and reduced expression of SIX3, the p53 mutant U251 cells were more sensitive to VX680 treatment, compared with the p53 wild-type U87 cells (Fig. 7b).

Three primary human patient-derived astrocytoma lines were cultured, and the expression of SIX3, AURKA, and AURKB was examined (Fig. 7c). Mutations in p53 coding regions were determined by sequencing of polymerase chain reaction products. G20151124B and 20151124C cells (mutant p53) with a low SIX3 expression were more sensitive to VX680 treatment compared with G20151114 cells with a high SIX3 expression (wild-type p53) (Fig. 7d). Treatment with two other aurora kinase inhibitors, AZD1152 and MLN8237, demonstrated similar results (Fig. 7d).

The above data indicate that although overexpression of SIX3 cannot enhance the sensitivity of astrocytoma cells to aurora kinase inhibitor treatment, SIX3 expression could be a more potential biomarker to evaluate the use of these agents in astrocytoma therapy. Astrocytoma patients with a low expression of SIX3 and mutant p53 are more sensitive to treatment with aurora kinase inhibitors.

## Discussion

Previous studies have demonstrated that SIX3 recognizes both TGATAC and ATTA/TAAT motifs, acting as a transcriptional activator or suppressor [23, 30]. In the forebrain, SIX3 binds to the conserved enhancer-2 upstream of sonic hedgehog (SHH) to activate SHH gene expression [51]. SIX3 has also been shown to bind to SIX3-responsive elements in the Wnt8b locus or the promoter of Wnt family member 1 (WNT1), leading to the repression of Wnt family member 8B (WNT8B) or WNT1 expression, respectively, promoting eye and forebrain development [29, 31]. In addition, a non-transcriptional function of SIX3 has been shown in which SIX3 directly binds with geminin, inhibiting its ability to sequester Cdt1, thereby promoting the proliferation of retinal precursor cells [52]. Here, we identified for the first time that SIX3 binds to the promoter regions of AURKA and AURKB and confirmed SIX3 as a crucial repressor that suppresses the transcription of AURKA and AURKB. Moreover, SIX3 also indirectly regulates AURKA and AURKB by suppressing the expression of the transcriptional activators of these aurora kinases, E2F1 and c-Myc (Additional file 1: Figure S14).

Though SIX3 is widely expressed in the central nervous system, its role in brain tumors has not been well studied. Our previous research characterized SIX3 as a hypermethylated gene in astrocytoma [38]. In this study, we further revealed SIX3 as an astrocytoma suppressor gene, which inhibits the tumorigenesis of astrocytoma. By targeting two mitotic mediators, AURKA and AURKB, SIX3 reduces the rate of numerical and structural centrosomal aberrations, preserving the correct separation of chromosomes. In vivo and in vitro empirical evidence confirmed that SIX3 reversed the malignant phenotype of astrocytoma cells. It has been reported that disruption expression or kinase activity of AURKA or AURKB in cancer cells impairs mitotic progression and results in G2/M phase arrest [5]. Here, we found that knockdown of AURKA in p53 mutant astrocytoma cells led to G1 phase arrest. This result is consistent with previous work that showed knockdown of AURKA in a p53 null cell line-induced G1 phase arrest [53]. Together, these data suggest that p53 status is critical for the regulation of the cell cycle by AURKA. While overexpression of SIX3 led to S phase arrest, we further confirmed that this effect of SIX3 was independent of AURKA and AURKB. Cyclin A plays multiple roles in cell cycle regulation by interacting with CDK1 and CDK2 [54]. Previous studies have revealed that cyclin A/CDK complex initiates DNA replication in S phase [54, 55]. Other studies also reported that overexpression or aberrant activation of cyclin A impairs replication origin firing and leads to prolonged S phase progression [56]. We found that SIX3 induced cyclin A, together with other cell cycle regulators E2F1 and p27, which may partially explain the effect of SIX3 on cell cycle regulation. Interestingly, we found that SIX3 decreased p21 expression in p53 mutant astrocytoma cells (Fig. 4a, c). As p21 has been confirmed to exert an oncogenic role in a p53 independent manner [57, 58], further functional studies on cell cycle by SIX3 are warranted. SIX3 binds with geminin to initiate DNA replication, activates the p53/p21 pathway, and represses AURKA and AURKB to maintain genomic stability. Another member of the Six family, SIX1, has been shown to play a role in oncogenic transformation, and recent studies found that the knockdown of SIX1 in breast cancer reduced genomic instability [25, 59].

Because of differing localizations and functions of AURKA and AURKB, interaction between these two aurora kinases has rarely been investigated [6, 7]. Here, we observed the physical interaction between AURKA and AURKB for the first time and confirmed that the interaction protected them from proteasomal degradation. With the increase in our knowledge of aurora kinases, nuclear AURKA has been found to bind to hnRNP K, acting as a transactivating factor to activate the expression of MYC and promoting breast cancer [14]. More studies on the interaction between AURKA and AURKB will help us to better understand the mechanism regulating the expression and activity of aurora kinases. To date, inhibitors targeting AURKA or AURKB have been designed to block the kinase activity of these kinases [16]. Drug resistance to kinase-activity inhibition and severe side effects including bone marrow suppression can occur, contributing to decreased benefit in cancer patients and failure or suspension of clinical trials [17, 47]. Our new findings strongly support an alternative strategy for therapeutic development by blocking the interactions between AURKA and AURKB and destroying their stability.

There are significant clinical implications of these findings. Targeting aurora kinases has been considered a potential anti-cancer strategy, and a series of aurora kinase inhibitors have been developed and subjected to early clinical trials [16, 60]. VX680 (tozasertib), a pan aurora kinase inhibitor, and MLN8237 (alisertib), a highly selective AURKA inhibitor, have been found to effectively suppress cell proliferation and prolong the survival of mice with intracranial glioblastoma xenografts [47, 48]. Expression of AURKA and c-Myc, together with p53 mutation, have been confirmed as biomarkers that govern the response to aurora kinase inhibitors [49, 50]. Data from a clinical trial of MLN8237 showed that AURKA expression may not be a significant factor, leaving a clear need for other potentially predictive biomarkers [61]. We found that high SIX3 expression in astrocytoma resulted in low sensitivity to aurora kinase inhibitors, and this may partially be due to low expression of AURKA and AURKB [49]. Astrocytoma patients with a low expression of SIX3 accompanied by mutant p53 are more sensitive to treatment with aurora kinase inhibitors. Thus, we propose that SIX3 may be a more plausible biomarker to evaluate the use of these agents.

## Conclusions

We demonstrate that in astrocytoma, SIX3 acts as a tumor suppressor gene by transcriptionally repressing AURKA and AURKB but does not affect the interaction of AURKA and AURKB. SIX3 silencing results in high expression of AURKA and AURKB in astrocytoma. We also find that the interaction between AURKA and AURKB aids in stabilization, partly maintaining the state of high expression and activity of AURKA and AURKB. Additionally, SIX3 increases the p53 activity and expression in post-translational level by negative regulation of AURKA and AURKB. Expression of SIX3 in astrocytoma patients correlates with sensitivity to treatment with aurora kinase inhibitors.

## Methods

### Chemicals and antibodies

VX680 (Tozasertib) (cat.M1881), MLN8237 (Alisertib) (cat.M1752), AZD1152-HQPA (M1663), and MG132 (cat.M1902) were purchased from Abmole. Cycloheximide (R750107) was purchased from Sigma-Aldrich. The following primary antibodies were used: AURKA (mouse, Cell Signaling, 12100, WB 1:1000; IHC 1:200; IP 1:250), AURKB (rabbit, Cell Signaling, 3094, WB 1:1000; IHC 1:200; IP 1:200), c-Myc (rabbit, Cell Signaling, 13987, WB 1:1000), cyclin A (mouse, Upstate, 12946, WB 1:1000), E2F1 (mouse, Merck Millipore 05-379, WB 1:1000, ChIP 1:250), Flag (mouse, Sigma-Aldrich, F1804, WB 1:2000, ChIP 1:250, IP 1:250), GAPDH (mouse, Sangon, D190090, WB 1:5000), GFP (rabbit, Beyotime, AG279, WB 1:500, IP 1:200), H3 (rabbit, Beyotime, AH433, WB 1:500), p21 (rabbit, Proteintech, 10355-1-AP, WB 1:1000), p27 (rabbit, Santa Cruz, SC-528, WB 1:200), p53 (mouse, Active Motif, 39739, WB 1:1000, ChIP 1:250), pericentrin (rabbit, Abcam, ab4448, IF 1:200), phospho-aurora A (Thr288)/aurora B (Thr232)/aurora C (Thr198) (rabbit, Cell Signaling, 2914, WB 1:1000), phospho-histone H3 (ser10) (rabbit, Santa Cruz, SC-8656, WB 1:200), SIX3 (rabbit, Santa Cruz, SC-134776, WB 1:200, IHC 1:100), and alpha-tubulin (mouse, Proteintech,66031-1-Ig, WB 1:5000, IF 1:500).

### Astrocytoma tissues and cell lines

Primary astrocytoma samples and matched clinical data were obtained from the Department of Neurosurgery, the Xiangya Hospital, Central South University. All samples collected have the informed consent of the patients, and all experiments using human tissues were approved by the Joint Ethics Committee of the Central South University Health Authority. Astrocytoma cell lines U251, U87, and U118 were bought from cell banks of Chinese Academy of Sciences (Shanghai, China). All astrocytoma cell lines were subjected to STR (short tandem repeat) test in 2013, and the last test was completed in December 2016. Human normal astrocytes HEB were obtained from the Guangzhou Institutes of Biomedicine and Health, Chinese Academy of Sciences (Guangzhou, China) [62]. HEB, U251, and U118 were cultured in DMEM high-glucose medium with 10% FBS. While U87 cells were cultured in MEM medium with 10% FBS at 37 °C and 5% CO2 condition. SIX3 stably expressing U251 cells (U251-SIX3) and GFP stably expressing U251 cells (U251-CON) were constructed with lenti-virus and cultured in the DMEM high-glucose medium with 10% FBS and 100 ng/ml Blasticidin S hydrochloride (Sigma-Aldrich, 15205).

### Primary astrocytoma cell culture

All samples were collected with informed consent from the patients, and all experiments using human tissues were approved by the Joint Ethics Committee of the Central South University Health Authority. Primary astrocytoma samples were minced with GentleMACS Dissociator (Miltenyi Biotec) and digested with 0.25% trypsin at 37 °C for 30 min. Digestion was stopped by adding trypsin inhibitor, and the digested tissues were passed through 40 μm nylon cell strainers (Corning, 352340) to get single-cell suspensions. Cells were cultured in DMEM/F12 containing 10% FBS. Astrocytoma cells were tested with GFAP and nestin staining and subcutaneous implantation in nude mice.

### Plasmids

SIX3 was amplified from HEB cells and inserted into pEGFP-C1, p3xFLAG-CMV-10, and plenti6v5dtopo plasmids. AURKA and AURKB were purchased from Genechem. AURKA were fused into pEGFP-C1 and p3xFLAG-CMV-10. AURKB were fused into pDsRed1-N1, pEGFP-N1, and p3xFLAG-CMV-10. Promoters of AURKA and AURKB were amplified from HEB cells and inserted into the pGL3-Enhancer vector. P53 luciferase reporter and pEGFP-C1-p53 were gifts from Prof. Ming Zhou (Cancer Research Institute, Central South University).

### RNA interference

The target sequences of SIX3 siRNAs (GenePharma) were as follows: (1) 5′-GCCAAACTTCGCCGATTCT-3′ and (2) 5′-ATCCTTGAGAACCACAAGT-3′. The target sequences of AURKA siRNAs (GenePharma) were as follows: (1) 5′-GGGTTTGTTGTCAACCAAT-3′ and (2) 5′-ATGCCCTGTCTTACTGTCA-3′. The target sequences of AURKB siRNAs (GenePharma) were as follows: 5′-ACATCCTGCGTCTCTACAA-3′ and 5′-GGAGGATCTACTTGATTCT-3′. The negative control siRNA sequence was 5′UUCUCCGAACGUGUCACGUTT-3′.

### Transient transfection and lenti-virus infection

Transient transfection of small interference RNA (siRNAs) and plasmids was followed by manufacture’s manual (Lipofectamine 3000 Reagent, Thermo Fisher, L3000015). The lenti-virus system purchased from Invitrogen (Thermo Fisher) contained four plasmids: plenti6v5dtopo, plp1, plp2, and plpvsvg. GFP-tagged SIX3 were conducted into plenti6v5dtopo; 8 μg plenti6v5dtopo, 5.8 μg plp1, 2.4 μg plp2, and 5.8 μg plpvsvg were transfected into 100 mm plate 293FT cells, and virus were harvested after 48 h. We added the virus to U251 cells and sorted with flow cytometry. Then, the cells were cultured in the medium containing 100 ng/ml Blasticidin S hydrochloride (Sigma-Aldrich, 15205).

### Luciferase reporter assay

We transfected the firefly luciferase reporter plasmids together with SIX3 and pRL-SV40 (Renilla luciferase) plasmids into U251 cells. Luciferase activity was detected according to the manufacturer’s protocol (Promega, E1910). The ratio of firefly to renilla was used to analyze the relative luciferase activity.

### RNA isolation and quantitative real-time polymerase chain reaction (RT-qPCR)

RNA was prepared from astrocytoma samples or cells or tumors with the RNeasy kit (QIAGEN, 73143). cDNA was transcribed from 2 μg total RNA using RevertAid First Strand cDNA Synthesis Kit (Thermo Fisher, K1622). RT-qPCR was performed with the SYBR® Premix Ex TaqTM II kit (Takara, RR82LR). Gene expression was normalized to GAPDH. All primers are presented in Additional file 2.

### Western blotting

Details of Western blotting and IP were previously described [10, 14]. Whole cell lysates were prepared with RIPA buffer (50 mM Tris-HCL, pH = 7.4; 1 M NaCl; 1 mM EDTA; 0.1% SDS; 0.5% SDC) supplemented with protease inhibitor cocktail (Roche, 04693132001) and phosphatase inhibitor (Roche, 04906845001). Nuclear and cytoplasmic proteins were prepared with Nuclear Extract Kit (Thermo Fisher, 78833). Fifty microgram proteins were subjected to electrophoresis in different percentages of gel according to the molecular weight of detected proteins. 0.22 μm PVDF membrane (Merck Millipore, ISEQ00010) were used to transfer proteins.

### Co-immunoprecipitation

For endogenous AURKA and AURKB interaction, 10^7^ cells were prepared and lysed with GLB+ buffer (10 mM Tris-HCl pH 7.5; 300 mM NaCl; 10 mM EDTA; 0.5% Triton X-100) containing protease inhibitor cocktail (Roche) and phosphatase inhibitor (Roche) or prepared with Nuclear Extract Kit (Thermo Fisher). Two micrograms of antibodies was incubated with 500 μg cell lysate for 12 h at 4 °C. Then, the solution was incubated with Protein G beads (Thermo Scientific, 20399) for another 2 h at room temperature. After the beads were washed and boiled, the lysates were subjected to Western blotting. For detection of exogenous protein interaction, cells were transfected with plasmids for 48 h before lysis.

### Chromatin immunoprecipitation assay

Cells in 150 mm plates were cross-linked with 1% formaldehyde in medium for 10 min with gentle shaking. Before collecting the cells, we added 1/10 volume of 1.25 M glycine for 5 min and washed them with cold PBS with protease inhibitor cocktail (Roche). The cells were lysed with 5.0 ml of cell lysis buffer (50 mM Tris-HCL, pH = 7.4; 150 mM Nacl; 1 mM EDTA; 1 mM EGTA; 5 mM MgCl2; 0.5% Triton X-100; proteinase inhibitors and phosphorylation inhibitors) for 5 min on ice. The supernatant was disgarded after centrifugation for 5 min at 500 g, 4 °C. The nuclear pellet was resuspended in 1.0 ml of cell lysis buffer (this time switched to 1.5 ml tube). Then, we lysed the nuclear pellet with 300 μl SDS lysis buffer (1% SDS, 10 mM EDTA, 50 mM Tris-HCl, pH = 8.0) by gently vortexing and incubated the tubes on ice for half an hour. The nucleus lysates were sonicated at the power of 50 W for 20 min on ice and centrifuged at 13,000 rpm for 10 min at 4 °C. A portion (10%) of chromatin was set as input. Then, we added cell lysis buffer to 100 μl chromatin to bring the total volume to 1000 μl and mixed the solution with 2 μg antibody for precipitation. After rotation for 12 h, the solutions were incubated with 50 μl protein G agarose/salmon sperm DNA (Merck Millipore, 16-201) for another 2 h at room temperature. One hundred microliters of 10% chelex-100 (Bio-Rad, 1421253) was added to the beads, and the mixture was incubated at 99 °C for 10 min to reverse cross-link after washing the beads. The supernatant was subjected to analysis. The primers used for PCR are presented in Additional file 2.

### Immunofluorescence and confocal laser scanning microscopy

For the analysis of co-localization of AURKA and AURKB, HEK293 cells were transfected with GFP-tagged AURKA plasmid and RFP-tagged AURKB plasmid. After 48 h, cells were fixed with 4% paraformaldehyde for 15 min at room temperature. Then, the cells were stained with DAPI (Beyotime Biotechnology, C1002). Confocal analysis was performed on the UltraVIEW VoX system (PerkinElmer) according to the manufacturer’s instructions. For mitosis analysis, U251-CON and U251-SIX3 cells were arrested at M phase as described (cell synchronization and M phase cell harvest). The cells were fixed with 4% paraformaldehyde for 15 min at room temperature and subsequently permeabilized with 0.25% Triton X-100 in PBS for 15 min. Then, the cells were stained with anti-tubulin (Proteintech, 66031-1-Ig) and anti-pericentrin (Abcam, ab4448) antibodies for 12 h at 4 °C, followed by staining with Alexa Fluor® 488 and 594 conjugated antibodies (Thermo Fisher, A11029, A27016). Then, the cells were stained with DAPI, and images were captured with a fluorescence microscope (Olympus).

### Cell synchronization and M phase cell harvest

For G1/S phase cell, cells were treated with 2 mM thymidine (Sigma-Aldrich, T1895) for 18 h; then, we removed thymidine and added fresh media to culture for 9 h. And later, we added thymidine to the medium to a final concentration of 2 mM for another 15 h. Cells were released and collected for cell cycle analysis. U251-CON and U251-SIX3 cells were synchronizated by double thymidine block. After releasing for 8 h, 10 μM MG132 (Abmole, M1902) was added, and we collected the cells 2 h later. The collected cells were arrested at M phase.

### Flow cytometric analysis

Cell Cycle and Apoptosis Analysis Kit with propidium staining (Beyotime Biotechnology, C1052) was used for flow cytometric analysis. Cells were harvested by trypsinization, washed once in cold PBS, and then suspended with 70% ethanol, and the cells were fixed overnight with rotation. Before staining, the cells were washed with PBS. Next, the cells were incubated with staining buffer containing PI and RNase A in 37 °C water bath for half an hour and then analyzed with flow cytometry (BD Biosciences).

### Colony formation and CCK8 assay

For colony formation assay, 24 h after transfected with plasmid or siRNAs, cells were dissociated and counted. One thousand cells/well were seeded into six-well plates and cultured for about 1 month. The cells were fixed with 4% paraformaldehyde and stained with H&E. CCK8 assay was used to assess cell viability. Detailed procedures were followed as previously described [63].

### EdU incorporation assay and transwell assay

EdU incorporation assay was used to detect proliferation ability of astrocytoma cells, and detailed procedures were followed as previously described [64]. Transwell assay was used to assess invasive ability of astrocytoma cells, and detailed protocol was as previously described [63].

### Intracranial implantation mouse model

All animal experiments were approved by the Animal Care and Use Committee of Central South University. Mouse orthotopic xenograft model was performed as previously described [48]. Five-week male nude mice (BALB/c) were anesthetized with intraperitoneal sodium pentobarbital (40 mg/kg) and fitted into stereotactic frames. One-centimeter incision was made on the midline, and a 1-mm burr hole was drilled at AP = +1 mm and ML = −2.5 mm from the bregma at the right hemisphere. Five microliters of 10^6^ cell was infused into the brain at a depth of −3.5 mm from the dura, at a speed of 1 μl/min. Mice weight and survival was recorded daily. After the mice were sacrificed, the whole brains were fixed with 4% paraformaldehyde. H&E staining and immunohistochemistry were performed according to standard protocols.

### Statistical analysis

All the experiments were repeated at least three times, and the representative data were showed. The statistical analysis was performed using GraphPad Prism 5(GraphPad Software, Inc.) and SPSS version 17.0 (SPSS Inc.). The Pearson coefficient was used to assess correlations between the expression of SIX3, AURKA, and AURKB. Statistical analysis was performed with Student’s *t* test and ANOVA. A *p* value of <0.05 was considered to be statistically significant.

## Additional files

Additional file 1: Supplementary figures S1–S13. (PDF 1864 kb)

Additional file 2: Sequences of primers for RT-qPCR and ChIP-PCR. (XLSX 12 kb)

## Abbreviations

AURKA: Aurora kinase A
AURKB: Aurora kinase B
AURKC: Aurora kinase C
CDE: Cycle-dependent element
ChIP: Chromatin-immunoprecipitation
CHR: Cell cycle gene homology region
CIN: Chromosomal instability
CNS: Central nervous system
Co-IP: Co-immunoprecipitation
CSCs: Cancer stem cells
ENCODE: Encyclopedia of DNA Elements
GBM: Glioblastoma
GFP: Green fluorescent protein
REMBRANT: Repository for Molecular Brain Neoplasia Data
RFP: Red fluorescent protein
RT-qPCR: Quantitative real-time polymerase chain reaction
siRNA: Small interference RNA
SIX3: SIX homeobox 3
