# Supplementary material for: SIX3, a tumor suppressor, inhibits astrocytoma tumorigenesis by transcriptional repression of AURKA/B
Source: J Hematol Oncol. 2017 Jun 8;10:115. doi: 10.1186/s13045-017-0483-2 (PMC5465582; doi:10.1186/s13045-017-0483-2)
Supplement: Supplementary file 1 — Supplementary figures S1–S13. (PDF 1864 kb) [file 13045_2017_483_MOESM1_ESM.pdf]

SIX3，a tumor suppressor, inhibits astrocytoma tumorigenesis by transcriptional repression of AURKA/B

Zhibin Yu[1,2],Yingnan Sun[1], Xiaoling She[3], Zeyou Wang[3], Shuai Chen[1], Zhiyong Deng[1], Yan Zhang[1,2], Qiang Liu[4], Qing Liu[5], Chunhua Zhao[1], Peiyao Li[1,2], Changhong Liu[1,2], Jianbo Feng[1,2], Haijuan Fu[1,2], Guiyuan Li[1,2], Minghua Wu[1,2*]

1. Hunan Provincial Tumor Hospital and the Affiliated Tumor Hospital of Xiangya Medical School, Central South University, Changsha 410013, Hunan, China.

2. The Key Laboratory of Carcinogenesis of the Chinese Ministry of Health, The Key Laboratory of Carcinogenesis and Cancer Invasion of the Chinese Ministry of Education, Cancer Research Institute, Central South University, Changsha, Hunan 410008, China.

3. The Second Xiangya Hospital, Central South University, Changsha, Hunan 410011, China.

4. The Third Xiangya Hospital, Central South University, Changsha, Hunan, 410011, China.

5. The Xiangya Hospital, Central South University, Changsha, Hunan 410008, China.

* To whom correspondence should be addressed: Minghua Wu

E-mail: wuminghua554@aliyun.com

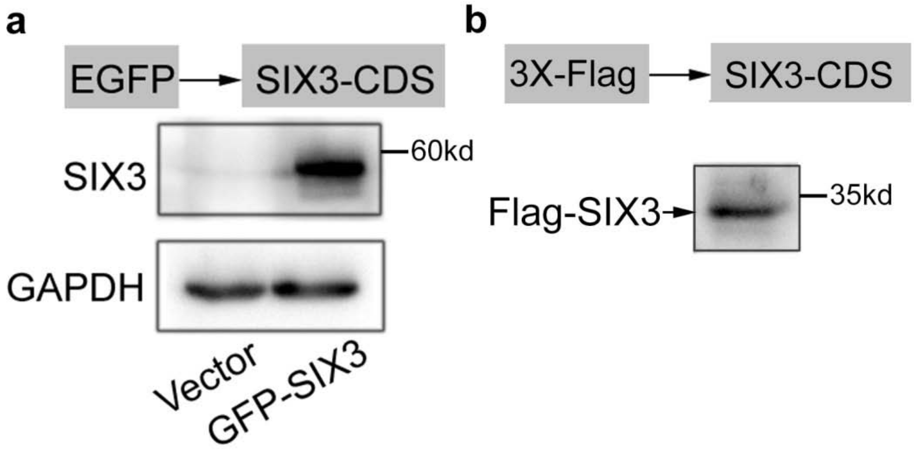

Figure S1. Construction of GFP- tagged SIX3**(a)** and Flag-tagged SIX3**(b)** over expression plasmid, and Confirmation with anti-GFP, anti-Flag antibody by western blotting. SIX3 CDS (Coding Sequence) was constructed at the C terminal of GFP or Flag.

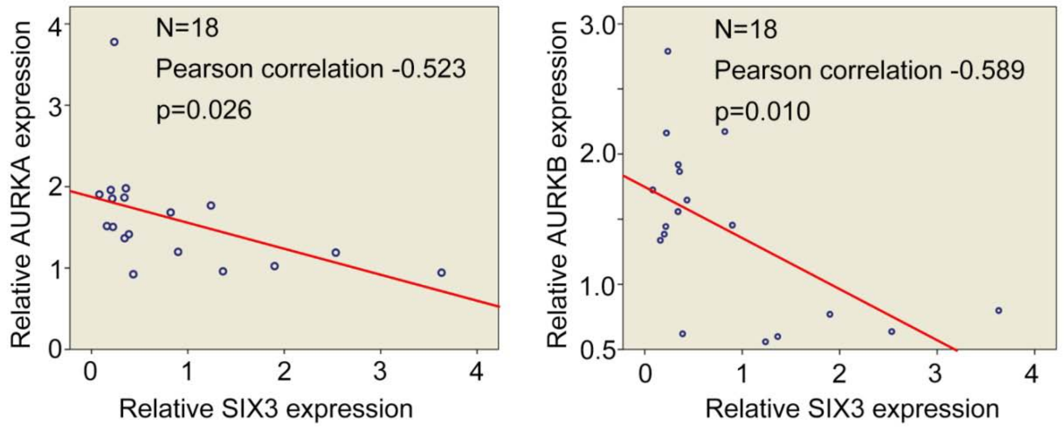

Figure S2. Correlation analysis of mRNA expression between SIX3 and AURKA(Left) , SIX and AURKB(Right) in 18 astrocytoma samples. The mRNA expression levels were detected with RT-qPCR, and normalized with GAPDH.

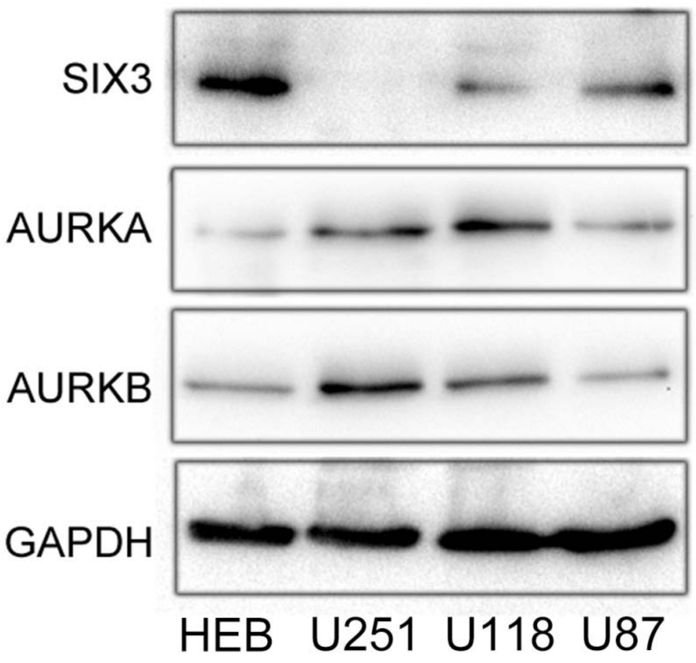

Figure S3. Western blotting analysis of expression of SIX3, AURKA and AURKB in normal astrocytes HEB and GBM cells U251, U118 and U87.

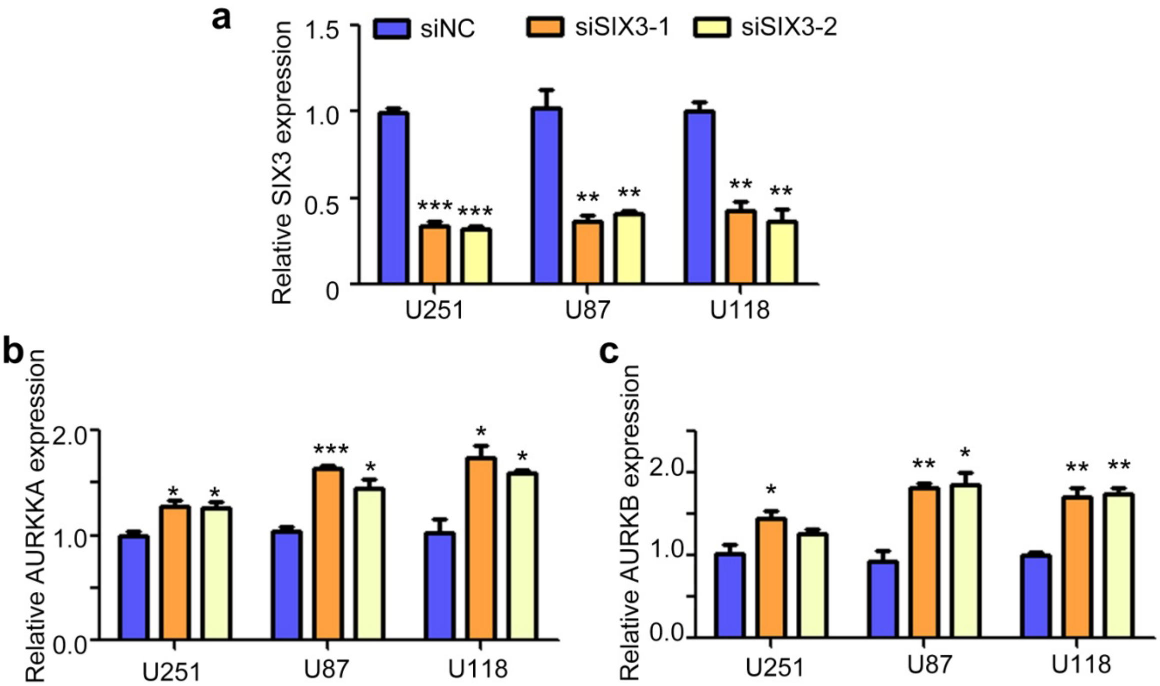

Figure S4. U251 ,U118 and U87 cells were transfected with two independent siRNAs. RT-qPCR analysis of mRNA expression of SIX3 **(a)**, AURKA **(b)** and AURKB **(c)** (normalized with GAPDH). Knockdown of SIX3 the increased expression of AURKA and AURKB.

(*,P<0.05;**,P<0.01;***,P<0.001)

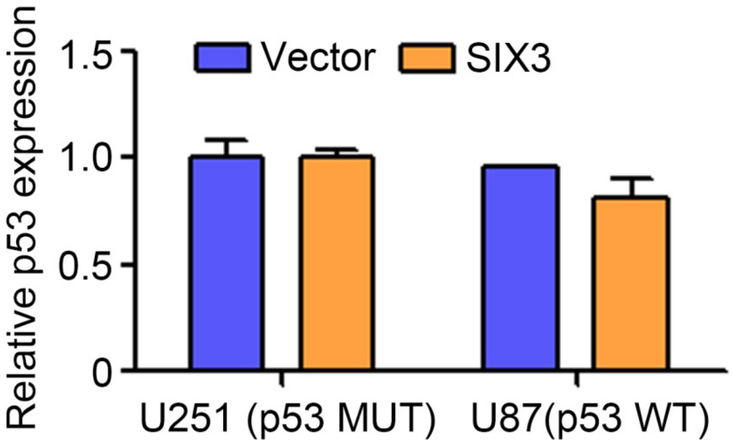

Figure S5. U251 and U87 cells were transfected with pEGFP-C1-SIX3. RT-qPCR analysis showed that SIX3 did not affect the mRNA expression of p53 in both U251 and U87.

(*,P<0.05;**,P<0.01;***,P<0.001)

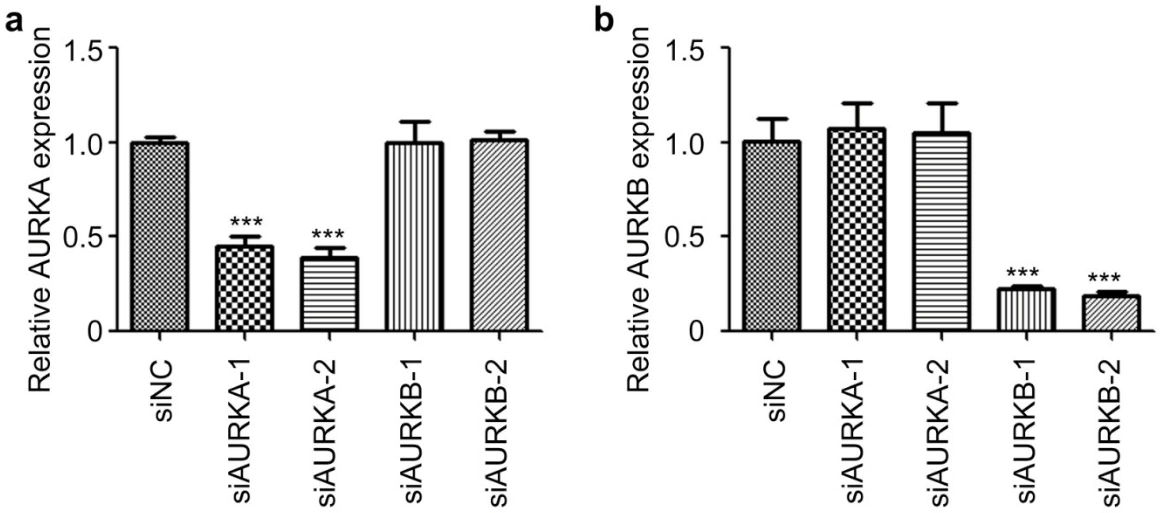

Figure S6. U251 cells were tranfected with siRNAs targeting AURKA or AURKB. RT-qPCR analysis showed that AURKA-knockdown did not affect the mRNA expression of AURKB, while AURKB-knockdown did not affect the mRNA expression of AURKA as well.

(*,P<0.05;**,P<0.01;***,P<0.001)

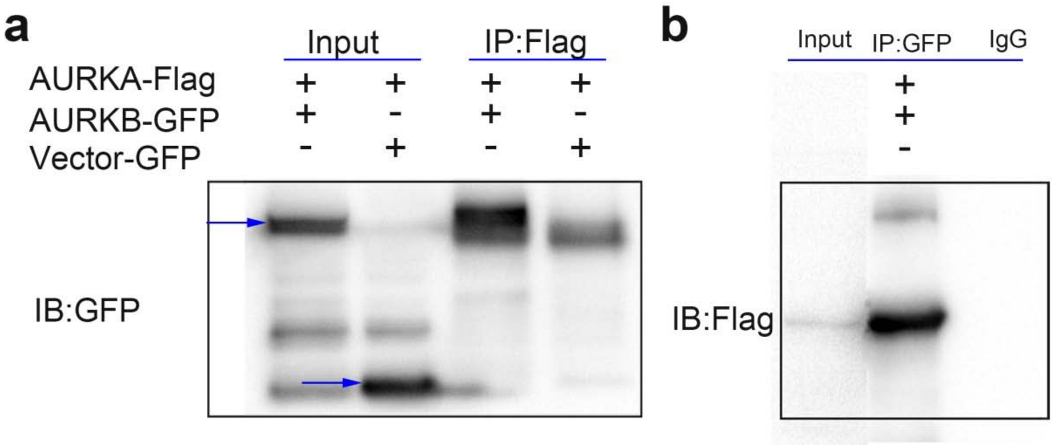

Figure S7. p3xFlag-CMV-10-AURKA and pEGFP-N1-AURKB were co-transfected into U251 cells. Co-IP and western blotting analysis showed the interaction between exogenous Flag tagged AURKA and GFP tagged AURKB.

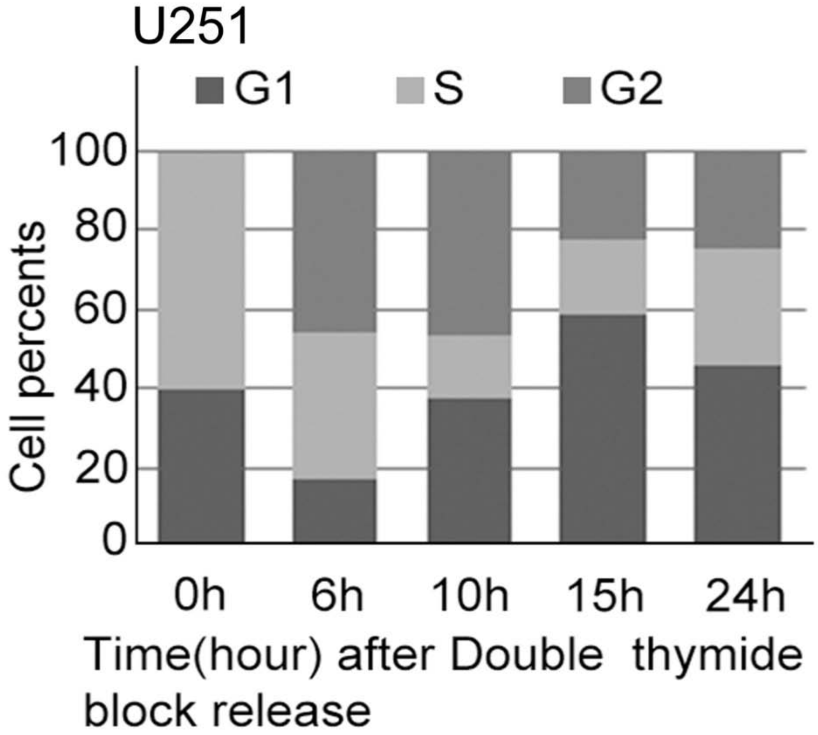

Figure S8. U251 cells was synchronized by double thymidine block. Flow cytometry analyzed cell cycle of U251 cells release for different times after double thymidine block. U251 cells enter G2/M phase in about 6 hours after release.

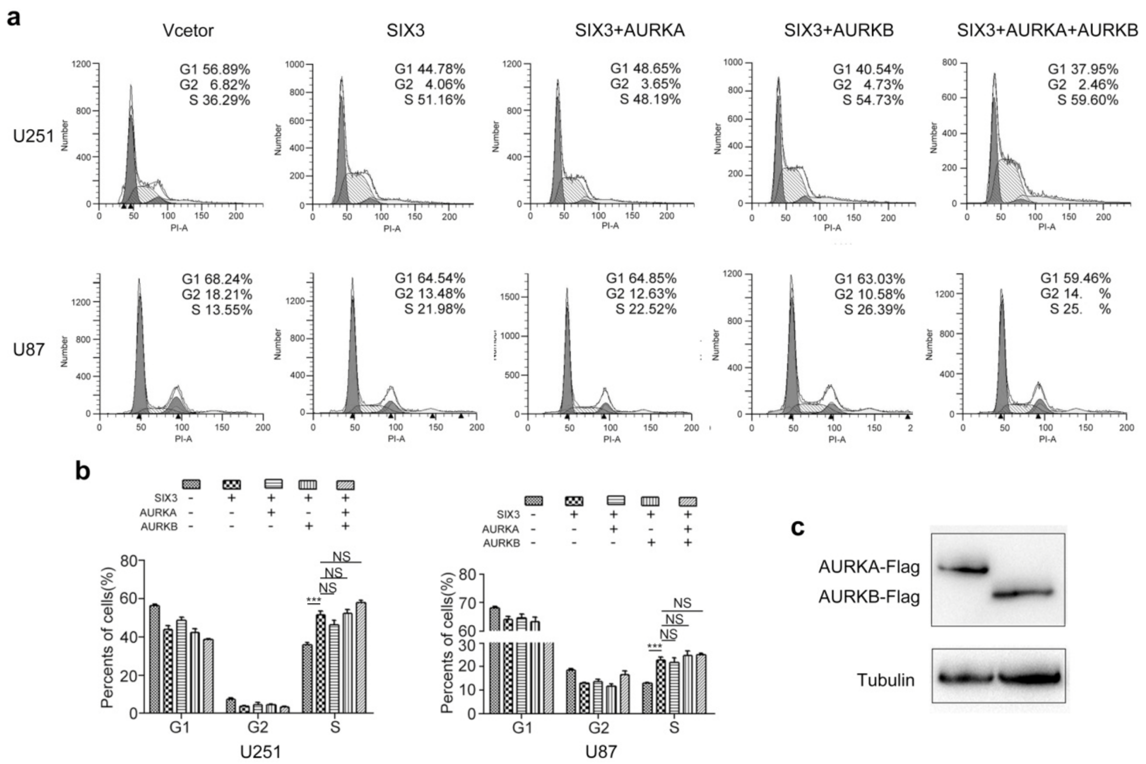

Figure S9. **(a)** and **(b)** U251 and U87 cells were transfected with pEGFP-C1-SIX3, pEGFP-C1-SIX3 & p3xFlag-CMV-10-AURKA, pEGFP-C1-SIX3 & p3xFlag-CMV-10-AURKB, pEGFP-C1-SIX3 & p3xFlag-CMV-10-AURKA & p3xFlag-CMV-10-AURKB. Flow cytometry analysis showed SIX3 induced S phase arrest, and rescue of AURKA and AURKB expression did not reverse SIX3 effect on cell cycle regulation both in U251 and U87 cells. **(c)** Confirmation of p3xFlag-CMV-10-AURKA and p3xFlag-CMV-10-AURKB with anti-Flag antibody by western blotting.

(*,P<0.05;**,P<0.01;***,P<0.001)

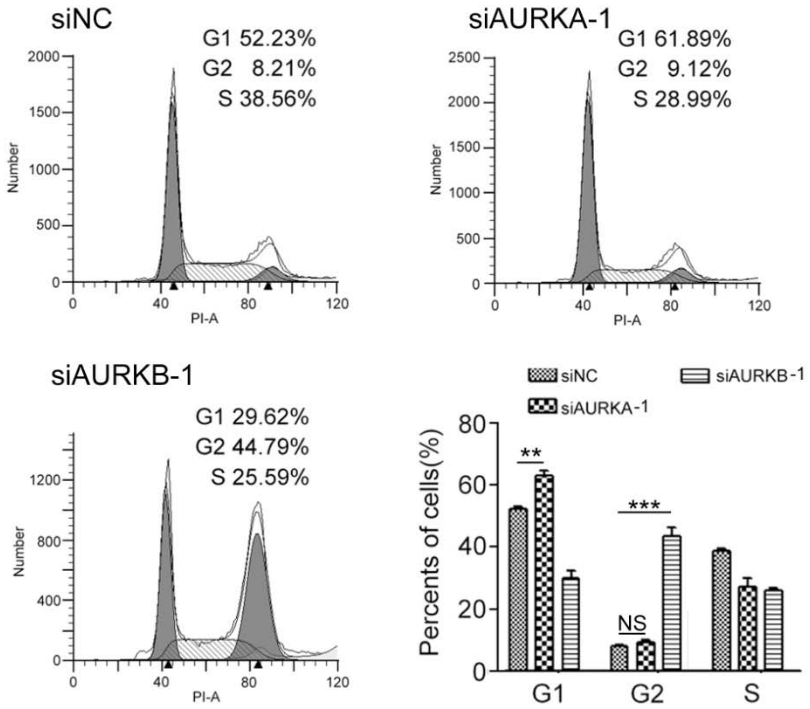

Figure S10. U251 cells were transfected with siRNAs targeting AURKA and AURKB. Flow cytometry analysis showed that knockdown of AURKA induced G1 phase arrest, and knockdown of AURKB induced G2/M arrest in p53 mutant U251 cells.

(*,P<0.05;**,P<0.01;***,P<0.001)

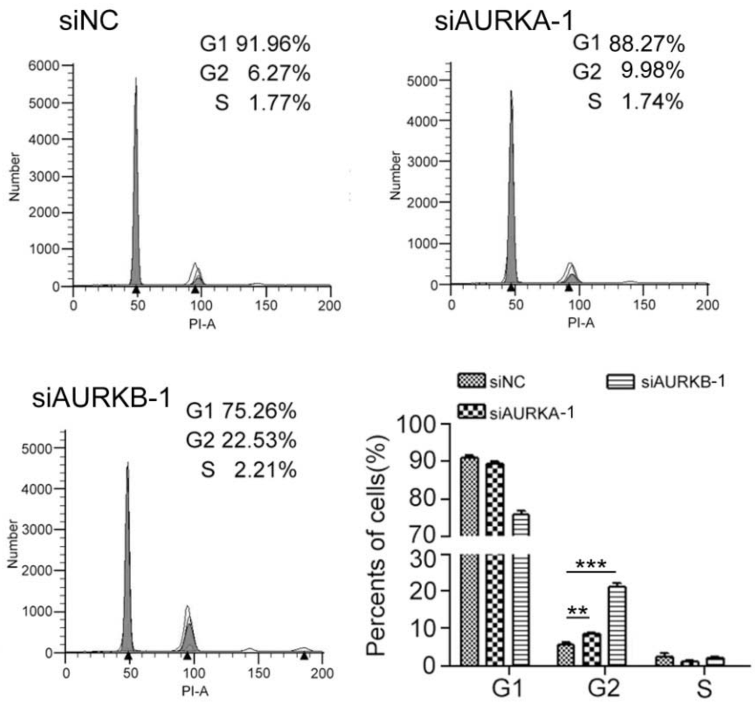

Figure S11. U87 cells were transfected with siRNAs targeting AURKA and AURKB. Flow cytometry analysis showed that knockdown of AURKA or AURKB both induced G2/M arrest in p53 wild-type U87 cells.

(*,P<0.05;**,P<0.01;***,P<0.001)

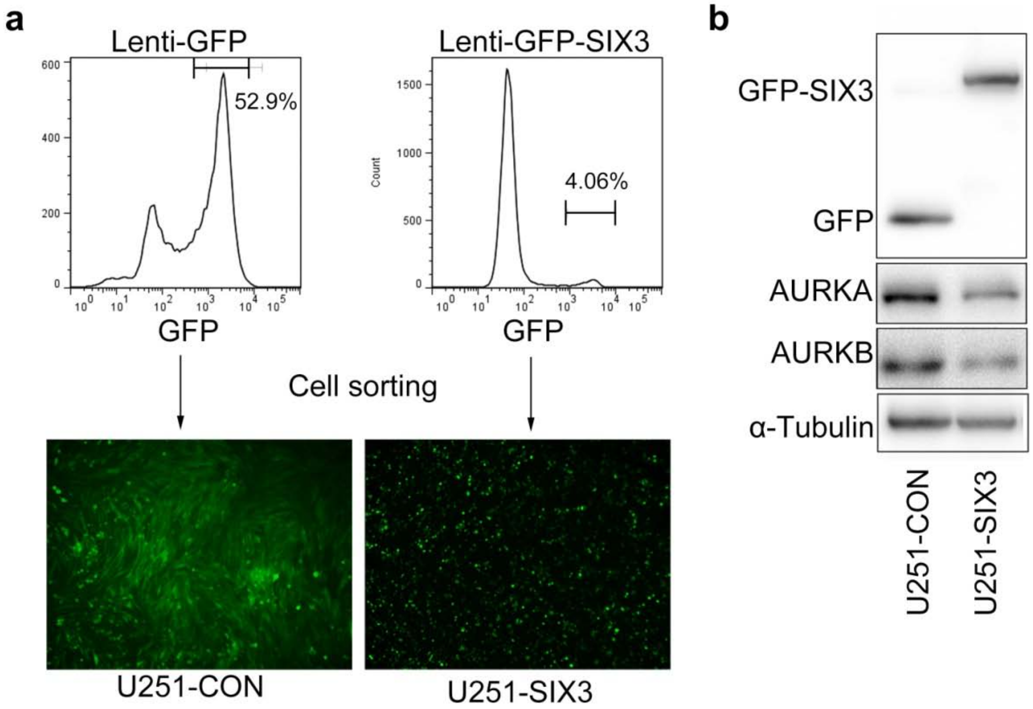

Figure S12. U251 cells were infected with lenti-virus Lenti-GFP-SIX3 and lenti-GFP. **(a)** Construction of GFP-SIX3 stably-expressing U251 cells (U251-SIX3) and GFP stably expressing U251 cells (U251-CON) using lenti-virus and sorted with flow cytometry. **(b)** Western blotting analysis showed U251-SIX3 cells expressed low levels of AURKA and AURKB compared with U251-CON cells.

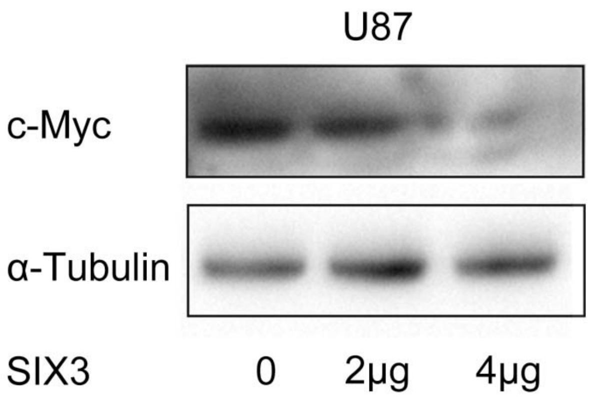

Figure S13. U87 cells were transfected with different dose of PEGFP-C1-SIX3. Western blotting analysis showed that SIX3 decreased the expression of c-Myc.
